# Contrasting Biogeographic and Diversification Patterns in Two Mediterranean-Type Ecosystems

**DOI:** 10.1371/journal.pone.0039377

**Published:** 2012-06-20

**Authors:** Sven Buerki, Sarah Jose, Shrirang R. Yadav, Peter Goldblatt, John C. Manning, Félix Forest

**Affiliations:** 1 Jodrell Laboratory, Royal Botanic Gardens, Kew, Richmond, Surrey, United Kingdom; 2 Department of Botany, Shivaji University, Kolhapur, India; 3 Missouri Botanical Garden, St. Louis, Missouri, United States of America; 4 Compton Herbarium, Kirstenbosch Research Centre, South African National Biodiversity Institute, Claremont, South Africa; University of Lausanne, Switzerland

## Abstract

The five Mediterranean regions of the world comprise almost 50,000 plant species (ca 20% of the known vascular plants) despite accounting for less than 5% of the world’s land surface. The ecology and evolutionary history of two of these regions, the Cape Floristic Region and the Mediterranean Basin, have been extensively investigated, but there have been few studies aimed at understanding the historical relationships between them. Here, we examine the biogeographic and diversification processes that shaped the evolution of plant diversity in the Cape and the Mediterranean Basin using a large plastid data set for the geophyte family Hyacinthaceae (comprising ca. 25% of the total diversity of the group), a group found mainly throughout Africa and Eurasia. Hyacinthaceae is a predominant group in the Cape and the Mediterranean Basin both in terms of number of species and their morphological and ecological variability. Using state-of-the-art methods in biogeography and diversification, we found that the Old World members of the family originated in sub-Saharan Africa at the Paleocene–Eocene boundary and that the two Mediterranean regions both have high diversification rates, but contrasting biogeographic histories. While the Cape diversity has been greatly influenced by its relationship with sub-Saharan Africa throughout the history of the family, the Mediterranean Basin had no connection with the latter after the onset of the Mediterranean climate in the region and the aridification of the Sahara. The Mediterranean Basin subsequently contributed significantly to the diversity of neighbouring areas, especially Northern Europe and the Middle East, whereas the Cape can be seen as a biogeographical cul-de-sac, with only a few dispersals toward sub-Saharan Africa. The understanding of the evolutionary history of these two important repositories of biodiversity would benefit from the application of the framework developed here to other groups of plants present in the two regions.

## Introduction

Although accounting for less than 5% of the world’s land surface, the five Mediterranean regions of the world are home to almost 50,000 species and as much as 20% of the known vascular plants [1], and are consequently considered by Conservation International to be among the 34 global biodiversity hotspots [2]. These five regions (the Cape Floristic Region, Southwest Australia, Central Chile, California Floristic Province, and the Mediterranean Basin) have warm and mainly dry summers contrasting with cool and wet winters. Before the Pliocene, the vegetation covering these regions was dominated by subtropical forest, which was gradually replaced by the current sclerophyllous shrublands: fynbos and renosterveld in the Cape, sclerophyllous forests and matorral in Chile, kwongan and mallee in Southwest Australia, chaparral in California, and matorral, maquis and garrigue in the Mediterranean Basin [1].

The remarkably rich biodiversity of the Cape flora has been extensively documented (e.g. [3]–[5]). Several evolutionary, ecological and environmental processes have been identified as the main causes responsible for this diversity, including low gene flow, climatic stability, topographic diversity, rainfall regime, edaphic factors and geomorphology, establishment of a fire regime, and pollinator shifts [3], [4], [6]–[11]. Although some of these factors have been more generally favoured than others (e.g. [12], [13]), none alone can explain the diversification patterns seen in many Cape-centred groups (i.e. “Cape clades”; [4]). With the analytical possibilities offered by molecular phylogenetics, the diversity found in the Cape is now known to have evolved from several independent radiations staggered over an extended period of time (since the early Miocene or even earlier), from various geographical sources, and involving a mixture of processes (e.g. [5], [7], [14]).

Similarly, the evolution of the Mediterranean Basin flora, with ca. 50% species endemism (comparable with many tropical areas [2]), has been widely studied and several major abiotic factors have been invoked to explain its species richness. It has been argued that the establishment of the Mediterranean climate (Middle to Late Miocene) coupled with fire regimes and the Messinian salinity crisis (which created the current insular system in combination with plate tectonic) are among the main factors explaining the rapid diversification of most of the herbaceous taxa found in the region [1], [15], [16]. In contrast, some have argued that most of the Mediterranean woody taxa originated in the late Cretaceous or early Tertiary, and are viewed as relics of previous evergreen and subtropical laurel forests [17], [18]. These taxa were presumably in place prior to the establishment of the Mediterranean climate and usually exhibit disjunct distribution patterns [17], [18].

**Figure 1:**
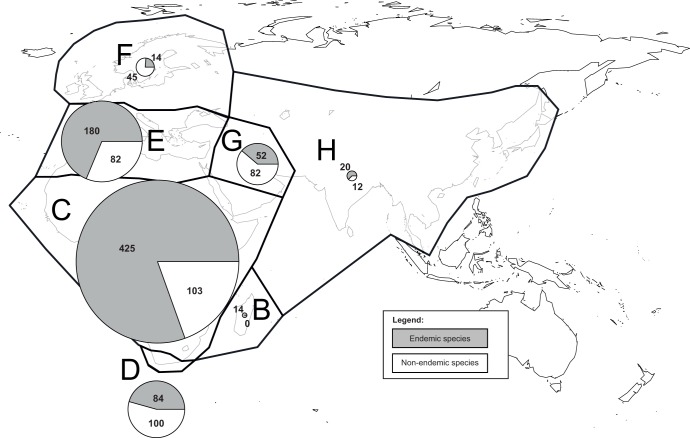
Area definition used to infer the biogeographical scenario of Hyacinthaceae. Species richness for each area is indicated. The numbers of species per area are based on Tables S1. B, Madagascar; C, sub-Saharan Africa; D, Cape of South Africa; E, Mediterranean Basin; F, Northern Europe; G, Middle East; H, Asia. Area A corresponds to America and is not represented in this figure.

The family Hyacinthaceae comprises approximately 1,000 species grouped in 35 genera [19], [20] and includes such well-known horticultural species such as bluebells (*Hyacinthoides* Heist. ex Fabr.), hyacinths (*Hyacinthus* Tourn. ex L.), squills (*Scilla* L.) and pineapple lilies (*Eucomis* L’Her.). These bulbous geophytes have very diverse floral and vegetative morphologies adapted primarily to arid, alpine, or seasonal climatic conditions [21], [22]. The family is almost exclusively Old World, where it is widely distributed throughout Africa and the Mediterranean Basin with some species in northern Europe, the Middle East and Asia; only one genus, *Oziroë* Raf. containing just five species, is found in the New World (endemic to western South America) (Fig. 1). Over half of the species of Hyacinthaceae are found in sub-Saharan Africa, with a major centre of diversity in southern Africa; the Mediterranean Basin is an important secondary centre of diversity (Fig. 1). The family has recently been included as the subfamily Scilloideae in an expanded Asparagaceae [23], [24] but because Hyacinthaceae comprise a monophyletic group within Asparagaceae sensu lato and has not received wide acceptance [19], [25], [26], we use the familial rank here. The taxonomy within the family has also been controversial as morphologically circumscribed genera often conflict with molecular systematic results (e.g. [19]). Generic delimitation in many cases remains in dispute, with some authors advocating larger genera and others a narrower circumscription (e.g. [19], [26]).

With its importance in the Cape and Mediterranean Basin both in terms of number of species and their morphological and ecological variability, Hyacinthaceae are an ideal case study to examine the patterns and processes that shaped the evolution of plant diversity in these two regions and their links to neighbouring biomes. Using the largest phylogenetic analysis for the family to date (ca. 25% of the species found in the family and three plastid markers), and state-of-the-art methods in analysis of biogeography and diversification, we address three questions in relation to the evolution of Hyacinthaceae, with a particular focus on their representation in the Cape and the Mediterranean Basin where more than 600 species are found (ca. 450 endemic) (Fig. 1). These questions are: 1) Where did the family originate and when?; it has been hypothesised that the family began to diversify after Gondwanaland split from Laurasia in the early Cretaceous [27]; 2) How different are the biogeographic and diversification patterns between the Cape and the Mediterranean Basin?; 3) Are the two regions sources of diversity (i.e. do taxa mainly disperse out of these regions), or are they repositories of diversity (i.e. do taxa mainly disperse into and accumulate in these regions). We examine the results of the analyses from the perspective of the evolution of these two global centres of plant biodiversity.

## Materials and Methods

### Taxon Sampling

A total of 256 species of Hyacinthaceae were sampled, representing all 35 recognized genera ( [20]; see Tables S1, S2). A total of 278 unpublished sequences were generated for the present study from samples newly extracted or already stored in the DNA Bank at the Royal Botanic Gardens, Kew (available online at: http://www.kew.org/data/dnaBank/; see Table S2 for voucher information and GenBank accession numbers). These novel data were supplemented by published sequences from previous studies (principally [19], [27], [28]; see Table S2). Outgroup taxa were selected based on a previous study of phylogenetic relationships within Asparagales [29], [30]. Members of Themidaceae (*Bessera* Schult.f, *Milla* Cav.), Agavaceae (*Camassia* Lindl., *Chlorogalum* (Lindl.) Kunth, *Hastingsia* S. Watson, *Hesperocallis* A. Gray) and Asparagaceae (*Asparagus* Tourn. ex. L.) were included as representatives of the families most closely related to Hyacinthaceae. *Asparagus* was defined as the most external outgroup taxon in all analyses.

### DNA Sequencing

Total genomic DNA was extracted from 0.03–0.3 g of silica gel-dried plant material or 0.25–1.25 g of fresh material using a modified version of the 2X CTAB method [31]. The total DNA was further purified for long-term storage in the DNA Bank at RBG Kew using a caesium chloride/ethidium bromide gradient (1.55 g/ml) followed by a dialysis procedure.

Three plastid regions were included in this analysis: the *trnL* intron and *trnL-F* intergenic spacer, forming together the *trnL-F* region, the barcoding portion of the *matK* gene, and the *rbcL* gene. The *trnL-F* region was amplified using primers c, d, e and f [32], the *matK* gene was amplified using the primers XF and 5R (see www.kew.org/barcoding), and the amplification of the *rbcL* gene was performed in two overlapping sections using the primers 1F and 724R (specific to monocots) for the first section and primers 636F and 1360R for the second section [33]–[35].

The amplification of all regions were conducted in 25 µl reactions, using 22.5 µl of Reddy PCR Master Mix (Thermo Fisher Scientific, Waltham, MA, USA), 0.9 µl of 0.4% bovine serum albumin, 0.3 µl of each primers (100 ng/µl) and 1 µl of template DNA. All amplifications were performed on a GeneAmp PCR System 9700 (Applied Biosystems, Foster City, California, USA). PCR conditions for *rbcL* and the *trnL-F* region were as follow: initial denaturation at 94°C for 2 min, followed by 28 cycles of 1 min at 94°C, 1 min at 50°C, 1.5 min at 72°C, ending with a single final elongation of 4 min at 72°C. Amplification of the *matK* gene follows the same programme except that it uses 32 cycles and an annealing temperature of 53°C. Cycle sequencing reactions were performed in 10 µl volume reactions containing 30–40 ng of PCR products, 0.5 µl BigDye® Terminator Cycle Sequencing kit (version 3.1; Applied Biosystems) and the same primers as for PCR. Cycle sequencing products were purified following the manufacturer’s protocols and complementary strands were sequenced on an ABI 3730 automated sequencer.

### Alignment and Phylogenetic Analyses

Sequencher 4.5 (Gene Codes Corp., Ann Arbor, Michigan, U.S.A.) was used to assemble complementary strands and verify software base-calling. The alignment of all DNA regions produced as part of this study and obtained from GenBank (see Table S2) was performed by eye in the Macintosh version of PAUP* (version 4.0b10; [36]).

Phylogenetic relationships were reconstructed using a Bayesian Markov Chain Monte Carlo (MCMC) approach as implemented in the software MrBayes, version 3.1.2 [37]. Since this study employs an expanded version of previous datasets, based on the same DNA regions, and all from the plastid genome, only a combined partitioned Bayesian MCMC analysis was conducted. In this analysis, each locus was allowed to have partition-specific model parameters [37]. MrModeltest 2.2 [38] was used to determine the best-fit model of DNA substitution for each partition (*trnL-F*, *rbcL* and *matK*) using the Akaike Information Criterion [39]. The General Time Reversible (GTR) model with a proportion of invariable sites and a gamma shape to account for rate heterogeneity among sites (GTR+I+G) was chosen for each partition. Four Metropolis-coupled Markov chains with an incremental heating temperature of 0.2 were run for 30 million generations and sampled every 1000^th^ generation. The analysis was repeated three times, starting from random trees, and performed on the Bioportal cluster at the University of Oslo (http://www.bioportal.uio.no). The MCMC sampling was considered sufficient when the effective sampling size was higher than 200, as verified with Tracer v1.5 [40] and when the potential scale reduction factor index approached 1.0 (as recommended in the MrBayes manual). After a burn-in period of 5 million generations per run, the remaining trees were used to reconstruct a consensus tree (i.e. the “allcompat” consensus tree of MrBayes) and to obtain Bayesian posterior probabilities (BPP) for each node. The choice of an “allcompat” consensus tree over a more traditional majority-rule consensus tree was motivated by the need of a fully resolved phylogenetic tree as input for the biogeographic and diversification analyses (see below). We also performed, for comparison purposes, a maximum likelihood (ML) analysis as implemented in the software RAxML [41]. The analysis was conducted on the CIPRES portal (http://www.phylo.org/) with partitioned data, the GTRCAT model, and 1000 rapid bootstrap replicates followed by the search of the best-scoring ML tree.

### Molecular Dating Analysis

The same approach as in Buerki et al. [42] was applied to estimate divergence times within Hyacinthaceae using the penalized likelihood method (PL; [43]). To account for phylogenetic uncertainty in divergence time estimation, the PL method was performed over a set of randomly selected phylogenetic trees (N = 2000) from the Bayesian MCMC stationary distribution and subsequently summarized on the consensus tree. A cross-validation routine, as implemented in r8s v.1.71 [44], was performed on the consensus tree using the Truncated Newton algorithm to establish the optimal smoothing value for this data set (S = 10). The same smoothing value was used for all the randomly selected trees. Because of limitations related to the calibration of the analysis (see below), only one external outgroup was kept in the dataset (*Asparagus* sp.). This outgroup taxon was subsequently pruned prior to the estimation of divergence times, as required by r8s (see [44]). Finally, TreeAnnotator [45] was used to estimate mean values and 95% confidence intervals of age estimates for each node based on the 2000 PL-dated phylogenetic trees and represented on the consensus tree.

Initially, we also used a Bayesian relaxed clock approach (BEAST; [45]) to estimate the posterior probability distribution of nodal heights, but several attempts failed to reach convergence, most likely due to the size and complexity of the data. In order to test potential bias of the PL algorithm (i.e. rate autocorrelation between branches [43]), additional BEAST analyses were performed with fixed tree topologies, using 20 randomly selected trees from the subset of 2000 MrBayes trees and used for the PL analysis. We used a relaxed lognormal molecular clock, linked partitions using a GTR+G+I model and a Yule speciation model; the other priors were set to default [45]. A run of 20 million generations was conducted for each analysis and sampled every 1000^th^ generation. Mean values and 95% confidence intervals of age estimates for each node were subsequently calculated using TreeAnnotator [45] with a burn-in value set at five million generations. Resulting node age estimates were compared with those obtained from the PL analysis using a regression.

Calibration of molecular phylogenetic trees is generally better performed using the fossil record [46], but monocots generally do not fossilise well and Hyacinthaceae are no exception to this condition; fossils for this family, and the rest of order Asparagales, are sparse and equivocal [47]. Thus, a single secondary calibration point was used: the crown node of the family was constrained with a fixed age of 70.1 million years (Ma) based on a previous molecular estimate for this node obtained from a monocot-wide analysis [48]. For the BEAST analyses, a normal distribution was applied with the mean value fixed at 70.1 Ma and a standard deviation of one.

### Area Definitions and Biogeographic Inference

The distribution of species included in the study was determined primarily from the World Checklist of Selected Plant Families (WCSPF) [20] and the third level delimitation of the Biodiversity Information Standards (TDWG) geographical codes [49]. Eight areas were defined on paleogeographic and climatic evidence: A) South America; B) Madagascar; C) sub-Saharan Africa; D) Cape of South Africa; E) Mediterranean Basin; F) northern Europe; G) Middle East; and H) Asia (see Fig. 1). All of these areas are separated by physical barriers or climatological differences. Assignment of species found in Turkey and Saudi Arabia required additional information because these countries overlap two of the above areas as a consequence of their variable habitats (Mediterranean Basin/Middle East and Middle East/sub-Saharan Africa, respectively). The WCSPF [20] almost always listed either a specific locality within Turkey (e.g. *Pseudomuscari coeleste* is found in east Turkey) or listed a species in Mediterranean countries as well as in Middle East countries, so it could be listed as occurring in both areas (e.g. *Scilla hyacinthoides* is found from France to Iraq). Saudi Arabia was split between sub-Saharan Africa and the Middle East. The Red Sea separating Africa from the Arabian Peninsula is less than 20 miles across at its narrowest point, allowing relatively easy dispersal of certain plants. This country presents a gradual change in habitat types from northeast (Middle East area) to southwest (sub-Saharan Africa area). Likewise, distribution information from the WCSPF [20] allowed the assignment of species to either or both of these areas. The representative sampling of Hyacinthaceae (both in terms of species richness and distribution) used in this study (Fig. S1) allowed terminals to be scored according to the distribution of the species. Although some species are widespread (e.g. *Muscari neglectum* occurs from Austria to Pakistan), most of the species are restricted to one or two areas (only one species occurs in four areas).

The dispersal-extinction-cladogenesis (DEC) likelihood model implemented in Lagrange v.2.0.1 [50], [51] was used to investigate the biogeographic history of Hyacinthaceae. This method is a parametric, extended version of the dispersal-vicariance analysis [52] that estimates ancestral ranges, transition rates between ranges, and biogeographic scenarios of range inheritance for a group of taxa in a ML framework [53]. The Lagrange analysis was performed on the PL-dated allcompat consensus tree with the maximum number of areas at nodes constrained to three. Ancestral area reconstructions for each node were plotted on the consensus tree using pie charts and a contingency table summarizing the biogeographic scenario (i.e. dispersal and extinction events through time) was produced using a collection of R scripts (see [42] for details). The contingency table was obtained by recording the area with the highest relative probability per node together with its age and the branch length leading to its descendant node. The biogeographic scenario was subsequently inferred (i.e. the type and frequency of transition events between ancestral and descendants nodes in the phylogenetic tree) according to the *Q* matrix implemented in the DEC model [42], [50]. The biogeographic scenario was presented in two time slices: i) before 16 Ma (middle Miocene) and ii) from 16 Ma to present. The choice of this boundary is motivated by i) the Mid-Miocene Climatic Optimum (MMCO) [54] and ii) the development of seasonal regimes which eventually led to the establishment of the Mediterranean climate [55].

To investigate the putative effect of past climate change on the dispersal and extinction of Hyacinthaceae, a lineage-through-time (LTT) plot was produced that also included the number of dispersals (or extinctions) though time (as inferred above) and the variation of isotopic O_18_ composition in the last 60 Ma [54]. The variation of isotopic O_18_ composition was proven to be a good proxy of temperature variability through time [54]. The LTT plot was inferred from the PL-dated allcompat tree using the R package ape [56].

### Reciprocal Effects between Geographic Range Evolution and Diversification

To investigate the patterns of diversification between areas – especially within areas of Mediterranean climate, the Mediterranean Basin and the Cape region – the Geographic State Speciation and Extinction model (GeoSSE) was applied to our data set [57]. This method simultaneously features the characteristics of the constant-rates birth-death model with a three-state Markov model [57] and is implemented in the R package *diversitree* [58]. This likelihood-based approach allows the estimation of region-dependant rates of speciation, extinction and range evolution based on a fully resolved dated phylogenetic tree (here the PL-dated allcompat tree). Seven parameters can be estimated by the model: speciation within regions A (sA) and B (sB), between-region speciation (sAB), extinction from regions A (xA) and B (xB), dispersal from A to B (dA) and dispersal from B to A (dB) (see Fig. 1 in ref. [57]). A strong advantage of this model lies in its ability to account for incomplete taxon sampling within areas [59]. To take into account incomplete taxon sampling within areas, a list of all Hyacinthaceae species and their respective distribution (according to the eight areas) was obtained from the WCSPF website [20] to determine the number of endemic and non-endemic species per area and the percentage of missing taxa for each area (see Fig. S1 for more details). For each area, a ML parameter estimation and model comparison was conducted followed by Bayesian parameter estimation through MCMC [57]. To reduce the complexity of the analysis, two GeoSSE models – the full model and the model without between region speciation (sAB) – were estimated under a ML framework and compared using a likelihood ratio test as implemented in *diversitree* [58]. For all analyses, the model without sAB always better fitted the data suggesting that there are regional differences in diversification. Subsequently a MCMC approach was used to perform a Bayesian analysis based on the six-parameter GeoSSE model (incomplete taxon sampling was also considered). ML rate estimates were used as priors to seed the MCMC analysis. The MCMC was run for 20,000 generations with a burn-in period of 5,000 generations. Finally, posterior probability distributions for the GeoSSE parameters were summarized using functions implemented in *diversitree* [58]. These analyses were not applied to the South America and Madagascar areas because they harbour only endemic species and thus are not suitable for GeoSSE analyses.

## Results

Species richness in Hyacinthaceae is highest in sub-Saharan Africa (528 spp., 80.5% endemism) followed by the Mediterranean region (262 spp., 68.7% endemism), the Cape (184 spp., 45.6% endemism) and the Middle East (134 spp., 38.8% endemism) (Figure 1) (see also Fig. S1 for taxon sampling per area). All other areas have less than 100 species. All species occurring in South America and Madagascar are restricted to these areas.

Despite the fact that the topology was fixed in the BEAST analyses, the effective sample sizes of several parameters remain low (i.e. just below 100). Nevertheless, the PL and BEAST analyses provided similar values for node age estimates (Fig. S2). Thus, the estimates obtained from the PL analyses are used for subsequent analyses.

**Figure 2:**
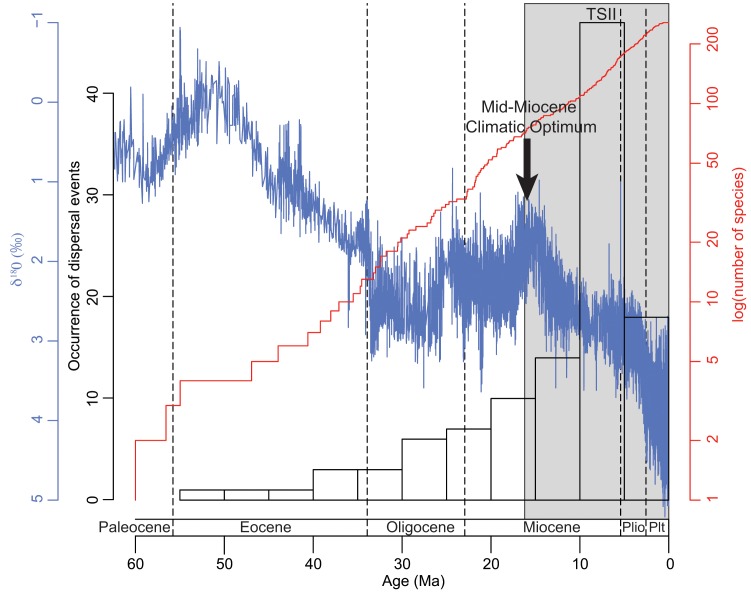
Occurrence of dispersal events through time for Hyacinthaceae inferred by Lagrange. The lineage through time plot (shown in red and based on the PL-dated consensus tree) is displayed with an estimation of climatic oscillations in blue (estimated from the variation of O_18_ concentration through time [54]).

Since we will not be giving detailed consideration to the historical biogeography of Hyacinthaceae in light of the current classification we provide detailed, dated phylogenetic trees with ancestral area reconstructions for the family and the subfamilies in Figure S3. The Bayesian and ML analyses produced very similar results and no significant discordance was noted between them; a comparison of support values (Bayesian posterior probabilities and bootstrap supports) for selected nodes is presented in Table S3. Figure 2 depicts the general dispersal pattern within Hyacinthaceae through time together with a LTT plot and the curve of O_18_ variation in the last 60 Ma (see Fig. S4 for extinction estimates). Although based on a fraction of the total number of taxa comprised in the family, one could extrapolate from this LTT plot that species in Hyacinthaceae have accumulated relatively constantly throughout the history of the group (Fig 2). A shift in the number of estimated dispersal and extinction events occurs shortly after the MMCO, with the highest number of dispersals estimated at the boundary between the Miocene and the Pliocene (Fig. 2).

**Figure 3:**
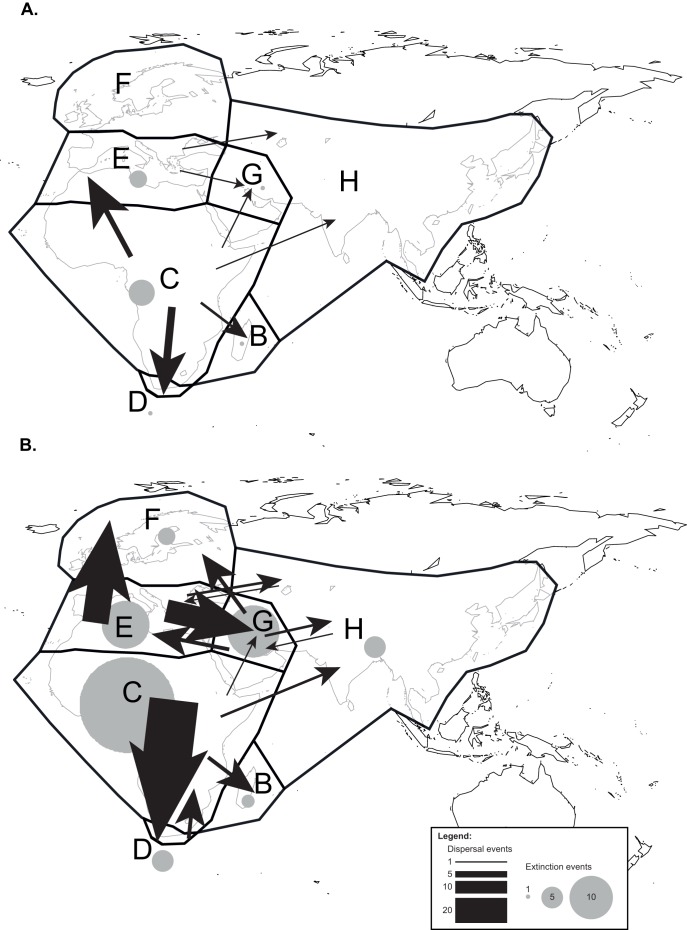
Biogeographical scenario of Hyacinthaceae inferred by the Lagrange analysis. Results are shown for before (A) and after (B) the Mid-Miocene Climate Optimum (16 Ma). Arrows depict dispersal events, whereas circles represent extinction events as estimated by the DEC model. See Fig. 1 for the abbreviations of biogeographical areas.

The dispersal and extinction events inferred by Lagrange are reported in two time slices: from 70 Ma to 16 Ma, and from 16 Ma to present (Fig. 3; see above for explanation). Note that events depicted in Figure 3 exclude the root node of the family, which has been assigned South America/sub-Saharan Africa as ancestral area (see Fig. S3). It is not possible to make further inference on this optimization without a denser sampling of closely related families and a better supported sister relationship to Hyacinthaceae, so we concentrate hereafter on the ancestral area reconstruction within the clade comprising the Old World species of the family (i.e. all but the five species of *Oziroë*).

The deeper nodes in the family have been assigned sub-Saharan Africa as the ancestral area, thus inferring several dispersal events from this area to all other regions except Northern Europe before 16 Ma, with the Cape and Mediterranean Basin receiving by far the most migrants (six and four, respectively; Fig. 3A). Dispersals also occurred between the Mediterranean Basin and the Middle East and Asia, but these were only minor events (one each). Extinction events in this first time slice are few and mostly concentrated in sub-Saharan Africa and the Mediterranean Basin.

The scenario inferred in the second time slice (16 Ma to present; Fig. 3B) differs drastically from the first, not only in the numbers of events (99 dispersals in the second time slice vs. 16 events during the first time slice) but also in the types of patterns observed (Fig. 3B). The number of dispersals increases sharply shortly after the MMCO, which coincides with the onset of conditions favourable for the establishment of Mediterranean conditions (Fig. 2); inferred extinction events also increase suddenly (Fig. S4). Sub-Saharan Africa remains an important contributor to the diversity of neighbouring areas, although three main aspects are particularly noteworthy in comparison with observations over the first time slice. First, and most significantly, there is no dispersal event inferred between sub-Saharan Africa and the Mediterranean Basin, although all other areas continue to benefit as recipients from sub-Saharan Africa (except Northern Europe). Secondly, the Cape region remains by far the principal recipient from sub-Saharan Africa (39 out of 99 dispersal events). Thirdly, the only dispersals towards sub-Saharan Africa are from the Cape and they are few (three dispersals). Sub-Saharan Africa was not a recipient during the first time slice (Fig. 3A). The Mediterranean Basin is now also a major contributor to the diversity of its neighbours, particularly Northern Europe (F) and the Middle East, with 21 and 18 dispersal events respectively. The Middle East can be viewed as a major crossroad, acting as an important contributor as well as serving as a recipient; it has no equivalent among the other areas in that respect. The importance of sub-Saharan Africa as a contributor is balanced by the number of extinction events inferred for this region (22 extinctions inferred by Lagrange), by far the largest among all areas. The Mediterranean Basin and Middle East regions also demonstrate significant numbers of extinction events, with 11 and 12 inferred extinctions respectively (Fig. 3B).

**Figure 4:**
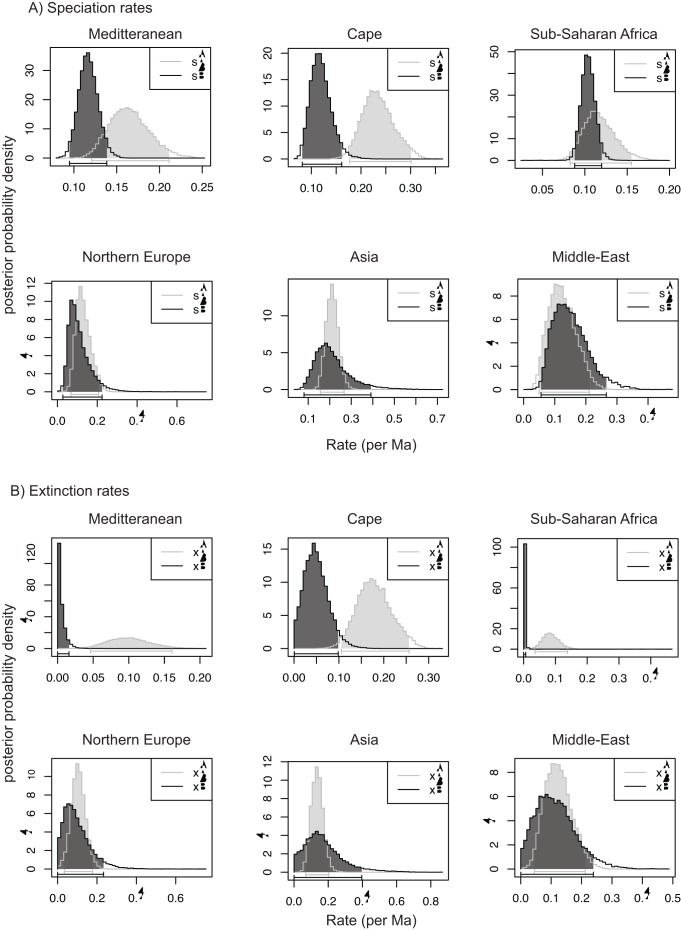
Posterior probability distributions for the speciation and extinction parameters. Speciation and extinction shown within a given region (sA and xA, respectively) and the remainder of the distributional range of Hyacinthaceae (sB and xB, respectively) for each area estimated by the GeoSSE analyses for the Hyacinthaceae dataset (see text for more details).

The GeoSSE analyses demonstrated higher speciation rates in the two Mediterranean climate regions (Mediterranean Basin and the Cape) than in the other regions (i.e. the rate of speciation within the other regions overlapped with the speciation rates outside of these areas, sA ∼ sB; Fig. 4A). Moreover, the Cape region has the highest speciation rate of all regions (Fig. 4A). Similar patterns are retrieved for extinction rates, with the Mediterranean Basin and Cape regions both showing higher extinction rates, together with sub-Saharan Africa, than the other areas studied (Fig. 4B). Finally, the GeoSSE model always inferred a higher rate of dispersal into the area of interest than out of it (dB>dA), even in the case of sub-Saharan Africa (data not shown).

## Discussion

### Biogeographic History of Hyacinthaceae

The biogeographical reconstruction presented here indicates that Hyacinthaceae originated in sub-Saharan Africa and began to diversify in the region around the Paleocene-Eocene boundary (Fig. S3); the deepest nodes in the Old World clade all received this single area as their most likely optimization. This scenario has been previously suggested by others, based either on a molecular phylogenetic analysis without an explicit biogeographical analysis [27] or with a less comprehensive taxonomic sampling and a single molecular marker [25]. Sub-Saharan Africa is the richest of all areas defined in the present study for Hyacinthaceae, with more than half of the species in the family found in the region (528 spp out of 999; Fig. 1). In addition of being the cradle of the family and the centre of species richness, sub-Saharan Africa is also the primary source of the species occurring in the two Mediterranean areas (Cape and Mediterranean Basin) and in Madagascar, and has contributed to the diversity found in the Middle East and Asia (Fig. 3).

One of the main differences in the subsequent history of the family in two Mediterranean regions lies in the disruption of the link between sub-Saharan Africa and the Mediterranean Basin sometime before the MMCO; all the dispersals from sub-Saharan Africa to the Mediterranean Basin took place before 20 Ma (Fig. 3). This disruption coincides with the aridification of northern Africa, which took place from the Late Miocene onwards [18] and eventually led to the formation of the Sahara Desert. This arid region acts as a physical barrier to dispersal events between sub-Saharan Africa and the Mediterranean Basin (Figs. 1, 3). In contrast, most of the dispersals between sub-Saharan Africa and the Cape (39 out of 45) took place after the MMCO, primarily between 10 Ma and 5 Ma (see Figs. 2, 3). This period coincides with the intensification of aridity in western South Africa and the development of a Mediterranean climate in the Cape (e.g. [60]). Although the current species richness in the two Mediterranean regions is similar and both exhibit the highest diversification rates among the regions examined here (Fig. 4), their evolutionary histories are strikingly different.

### Contrasts between the Mediterranean Regions

The high diversification rate found in the Mediterranean Basin is mainly the result of *in situ* speciation processes [61] (Fig. 3). This area was only colonized prior to the MMCO and further large-scale immigration events were prevented by physical barriers, notably the Sahara Desert (with the exception of three dispersals from the Middle East and one from Asia, all by widespread species; Fig. 3B). The most obvious triggers of this diversification are the establishment of the Mediterranean climate and concomitant fire regimes, as well as the Messinian salinity crisis and associated drier climates [1], [15], [55]. Several studies have reported increased diversification rates in herbaceous Mediterranean groups during this period (e.g. [62], and references therein). However, the influence of Quaternary climatic oscillations on the evolutionary processes responsible for the current species diversity should not be disregarded, especially in this region. It has been widely reported that these climatic fluctuations greatly influenced the speciation processes in most plants groups found in the region. The impact of the insular system in the Mediterranean on the diversification of bulbous plants, including Hyacinthaceae, remains to be fully explored, as has been done in other groups of the region [16], [63].

Although there is no evidence of dispersal events from the Mediterranean Basin into sub-Saharan Africa after the MMCO, the Mediterranean area is an important source area for colonization of Northern Europe (21 out of 24 dispersal events to this region) and has also contributed greatly to the diversity in the Middle East and, to a lesser extent, Asia (Fig. 3B). Most of the dispersals from the Mediterranean Basin to Northern Europe occurred within the last two million years, suggesting that the colonization of Northern Europe took place gradually through the Quaternary (Figs. 2, 3). This is consistent with a role of the Mediterranean Basin as a refugium during the Quaternary glaciations, as suggested by several authors (e.g. [64]–[66]). Our analyses also provide additional evidence for the importance of the Mediterranean Basin as a ‘tension zone’ for plant lineages of various biogeographical origins (see [61] and references therein) by virtue of its location at the crossroads between Europe, Asia and Africa.

In the southern Hemisphere, the high diversification rate found in the Cape is most likely linked to both the establishment of the Mediterranean climate in the region and a high immigration rate from sub-Saharan Africa. Just six dispersals were inferred from sub-Saharan Africa to the Cape before 16 Ma, whereas 39 dispersals were recorded by the Lagrange analysis in the last 16 Ma. The mean date of these latter dispersals is 5.6 Ma, corresponding to the establishment of the Mediterranean climate in this region (e.g. [60]; Figs. 3, 4). Unsurprisingly, given its position at the tip of the continent, the Cape represents a biogeographical cul-de-sac, and the region has not contributed significantly to the diversity of Hyacinthaceae in other areas, with the exception of a few dispersal events into sub-Saharan Africa, all by widespread species that originated in the Cape. Its geographic isolation probably also ensured that the Cape was not enriched by emigrants from areas other than sub-Saharan Africa (Fig. 3B).

High diversification rate of Hyacinthaceae in the Cape (Fig. 4) has now been demonstrated in several other plant taxa, including Iridaceae [67] and Proteaceae [68]. Commonly proposed drivers of this diversification include adaptation to frequent fires and nutrient-poor soils, low vagility, geographic isolation and edaphic specialisation, and relatively muted climatic oscillations. The relative frequency of extinction events in the flora is far less clear. Our analysis shows that the extinction rate in Hyacinthaceae is higher in the Cape than elsewhere, implying that species turnover is relatively high in the family compared to other regions. This result coincides with the high levels of ß- and γ-diversity that characterise the flora of the region [69], [70]. This contrasts with the recent tendency to link high species diversity in the region with low rates of extinction, ostensibly due to reduced competition from other lineages [68]. Lineage accumulation in the southwest corner of the African continent has been shown to be gradual in several groups (e.g. [71]), bolstering the conclusion that the high diversity of species is not predominantly the result of recent rapid radiations (although some groups do present this pattern; e.g. *Heliophila* [72]). Although we show that speciation rates in the Cape are undeniably higher than elsewhere, we also show the region is characterised by the highest extinction rate of all six regions. This result is difficult to reconcile with the proposition that the rich plant diversity of the Cape is due to a combination of high speciation rates and low extinction rates (e.g. [73]). Paradoxically, the large proportion of narrowly endemic species that characterises the Cape flora [70] is consistent with this pattern: narrow endemics are at greater risk of extinction from stochastic events than more widespread taxa. High rates of extinction are thus an inevitable corollary of high rates of evolution of narrow endemics.

### The Pivotal Importance of the Middle East Area

The Middle East area seems to have been a significant evolutionary crossroad between the northern hemisphere areas (Mediterranean Basin, Northern Europe and Asia; Fig. 3), especially after the MMCO. The Hyacinthaceae of the Middle East area, contrary to that of most of the other areas, shows a complex biogeographical history involving immigrations from various areas (sub-Saharan Africa, the Mediterranean Basin and Asia) and emigrations to several neighbouring areas (Fig. 3). No other area has as many interactions with its neighbours. The region harbours several lineages of different ages and geographic origins and may have played a key role as a refugium of phylogenetic diversity in the northern Hemisphere representatives, especially during periods of drastic climatic fluctuations.

### Conclusions and Perspectives

The combined approach of parametric biogeographic reconstruction, which factors in paleogeographic history and phylogenetic uncertainty, and the joint assessment of the effect of geographic range evolution and diversification, provides powerful tools to examine the patterns and processes responsible for the evolution of biodiversity. We have used these tools to shed light on the evolution of plant diversity in two of the most species-rich Mediterranean ecosystems, the Cape of South Africa and the Mediterranean Basin. Our analysis is based on a single family with a good representation in both Mediterranean ecosystems and a complete representation at the generic level. However, despite including more than 250 species, this data set covers only ca 25% of the species diversity of the family. Thus, conclusions drawn from the analyses presented here should be viewed cautiously, but provide nevertheless a valuable insight into the diversification of these unique biodiversity hotspots. Similar studies in additional groups are necessary to assess the general validity of the patterns uncovered here before they can be generalized to the diversification processes in these species-rich regions. Significantly, however, rates of extinction in the Cape are shown to be commensurate with rates of speciation, suggesting that the two processes may represent two sides of the same evolutionary coin in a region that is well known for its biological riches.

## Supporting Information

Figure S1 **Histogram summarising the Hyacinthaceae species sampling per area.**
(DOC)

Figure S2 **Correlation between PL and BEAST node age estimates based on 20 trees from the MrBayes analysis (see Material and Methods section for more details).** Regression lines (in grey) are provided for each tree.
(DOC)

Figure S3 **Biogeographical reconstruction of Hyacinthaceae using Lagrange and displayed on the PL-dated allcompat tree.** A) overview of the family with the 95% confidence intervals on divergence time estimates indicated on each node; B) subfamily Hyacinthoideae; C) subfamily Ornithogaloideae; D) subfamily Urginoideae; E) legend for ancestral areas.
(DOC)

Figure S4 **Occurrence of extinction events through time for Hyacinthaceae inferred by Lagrange.** The lineage through time plot (shown in red and based on the PL-dated consensus tree) is displayed with an estimation of climatic oscillations in blue (estimated from the variation of O_18_ concentration through time [54]).
(DOC)

Table S1 Classification and genera of Hyacinthaceae, with generic delimitation, distribution and species numbers.
(DOC)

Table S2 Voucher information and GenBank accession numbers for sequences used in this study.
(XLS)

Table S3 Divergence time estimates and associated confidence intervals for selected nodes.
(DOC)
