# Supplementary material for: Contrasting Biogeographic and Diversification Patterns in Two Mediterranean-Type Ecosystems
Source: PLoS One. 2012 Jun 20;7(6):e39377. doi: 10.1371/journal.pone.0039377 (PMC3379972; doi:10.1371/journal.pone.0039377)
Supplement: Figure S1 — Histogram summarising the Hyacinthaceae species sampling per area. (DOC) [file pone.0039377.s001.doc]

**Electronic Supplementary Material**

**Contrasting biogeographic and diversification patterns in two Mediterranean-type ecosystems**

**Sven BUERKI^1,5,6^, Sarah JOSE^1,5^, Shrirang R. YADAV^2^, Peter GOLDBLATT^3^, John C. MANNING^4^, Félix FOREST^1,6^**

^1^Jodrell Laboratory, Royal Botanic Gardens, Kew, Richmond, Surrey, TW9 3DS, United Kingdom.

^2^Department of Botany, Shivaji University, Kolhapur-416 004(MS), India.

^3^B.A. Krukoff Curator of African Botany, Missouri Botanical Garden, PO Box 299, St. Louis, MO ^6^3166-0299, U.S.A.

^4^Compton Herbarium, Kirstenbosch Research Centre, South African National Biodiversity Institute, Claremont 7735, South Africa.

^5^ These authors contributed equally to this work and are considered co-first authors

^6^ Authors for correspondence: [s.buerki@kew.org](mailto:s.buerki@kew.org); [f.forest@kew.org](mailto:f.forest@kew.org)

**Figure S1.** Histogram summarising the species sampling per area; the total number of species is presented as well as number of species sampled for each area.
