# Supplementary material for: Contrasting Biogeographic and Diversification Patterns in Two Mediterranean-Type Ecosystems
Source: PLoS One. 2012 Jun 20;7(6):e39377. doi: 10.1371/journal.pone.0039377 (PMC3379972; doi:10.1371/journal.pone.0039377)
Supplement: Figure S2 — Correlation between PL and BEAST node age estimates based on 20 trees from the MrBayes analysis (see Material and Methods section for more details). Regression lines (in grey) are provided for each tree. (DOC) [file pone.0039377.s002.doc]

**Electronic Supplementary Material**

**Contrasting biogeographic and diversification patterns in two Mediterranean-type ecosystems**

**Sven BUERKI^1,5,6^, Sarah JOSE^1,5^, Shrirang R. YADAV^2^, Peter GOLDBLATT^3^, John C. MANNING^4^, Félix FOREST^1,6^**

^1^Jodrell Laboratory, Royal Botanic Gardens, Kew, Richmond, Surrey, TW9 3DS, United Kingdom.

^2^Department of Botany, Shivaji University, Kolhapur-416 004(MS), India.

^3^B.A. Krukoff Curator of African Botany, Missouri Botanical Garden, PO Box 299, St. Louis, MO ^6^3166-0299, U.S.A.

^4^Compton Herbarium, Kirstenbosch Research Centre, South African National Biodiversity Institute, Claremont 7735, South Africa.

^5^ These authors contributed equally to this work and are considered co-first authors

^6^ Authors for correspondence: [s.buerki@kew.org](mailto:s.buerki@kew.org); [f.forest@kew.org](mailto:f.forest@kew.org)

**Fig. S2.** Correlation between PL and BEAST node age estimates based on 20 trees from the MrBayes analysis (see Material and Methods section for more details). Regression lines (in grey) are provided for each tree.

**
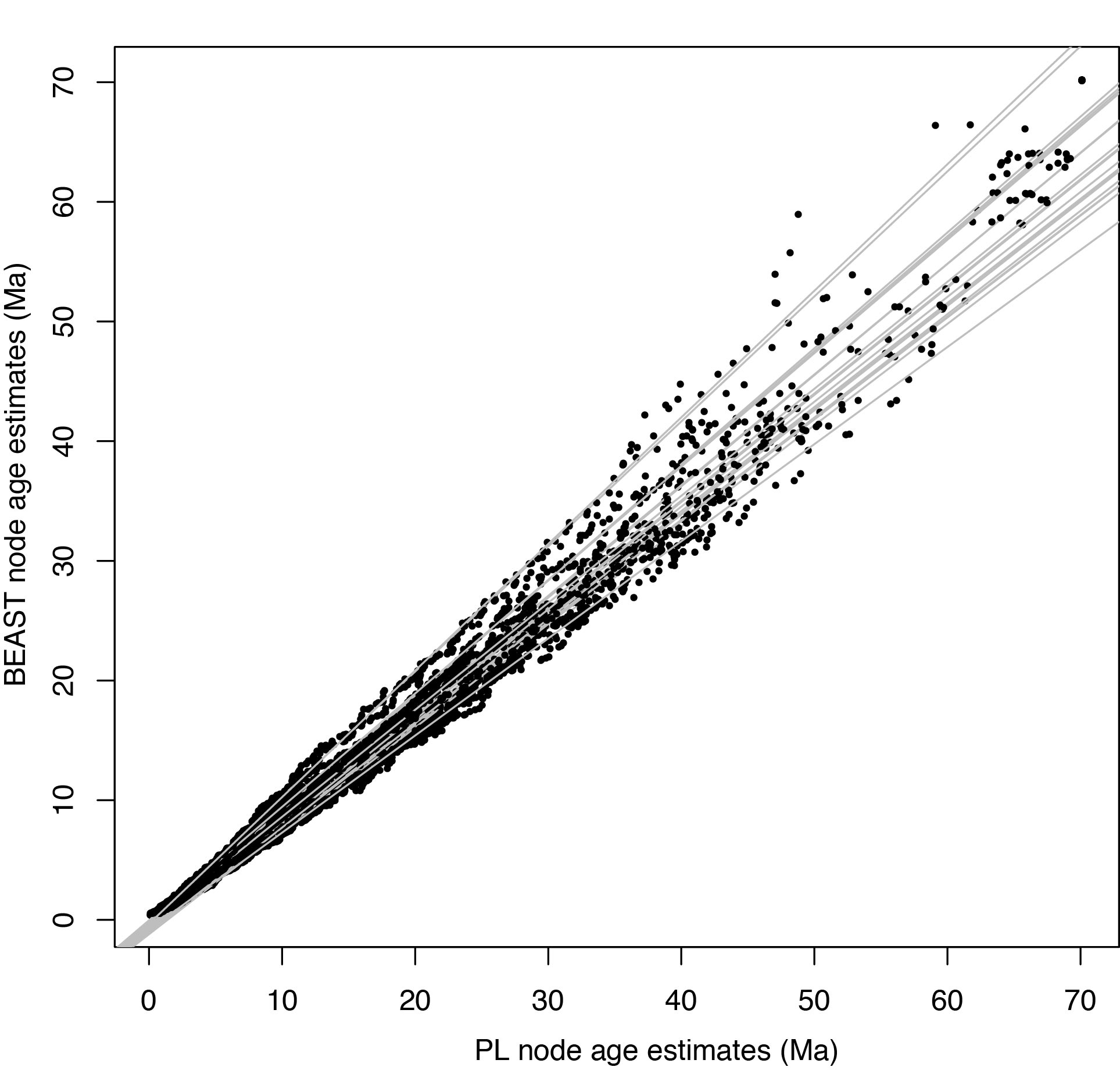
**
