# Supplementary material for: Contrasting Biogeographic and Diversification Patterns in Two Mediterranean-Type Ecosystems
Source: PLoS One. 2012 Jun 20;7(6):e39377. doi: 10.1371/journal.pone.0039377 (PMC3379972; doi:10.1371/journal.pone.0039377)
Supplement: Figure S3 — Biogeographical reconstruction of Hyacinthaceae using Lagrange and displayed on the PL-dated allcompat tree. A) overview of the family with the 95% confidence intervals on divergence time estimates indicated on each node; B) subfamily Hyacinthoideae; C) subfamily Ornithogaloideae; D) subfamily Urginoideae; E) legend for ancestral areas. (DOC) [file pone.0039377.s003.doc]

**Electronic Supplementary Material**

**Contrasting biogeographic and diversification patterns in two Mediterranean-type ecosystems**

**Sven BUERKI^1,5,6^, Sarah JOSE^1,5^, Shrirang R. YADAV^2^, Peter GOLDBLATT^3^, John C. MANNING^4^, Félix FOREST^1,6^**

^1^Jodrell Laboratory, Royal Botanic Gardens, Kew, Richmond, Surrey, TW9 3DS, United Kingdom.

^2^Department of Botany, Shivaji University, Kolhapur-416 004(MS), India.

^3^B.A. Krukoff Curator of African Botany, Missouri Botanical Garden, PO Box 299, St. Louis, MO ^6^3166-0299, U.S.A.

^4^Compton Herbarium, Kirstenbosch Research Centre, South African National Biodiversity Institute, Claremont 7735, South Africa.

^5^ These authors contributed equally to this work and are considered co-first authors

^6^ Authors for correspondence: [s.buerki@kew.org](mailto:s.buerki@kew.org); [f.forest@kew.org](mailto:f.forest@kew.org)

**Figure S3.** Lagrange biogeographic reconstruction of Hyacinthaceae displayed on the PL-dated allcompat tree. A) overview of the family with 95% PL intervals of confidence on nodes. Numbers on nodes refer to Table S3. The classification follows the World Checklist of Selected Plant Families. See E) for the legend of ancestral area reconstructions.

**Figure S3.** Biogeographic reconstruction on the PL-dated allcompat tree; B) focus on subfamily Hyacinthoideae. The classification follows the World Checklist of Selected Plant Families. See E) for the legend of ancestral area reconstructions.

**Figure S3.** Biogeographic reconstruction on the PL-dated allcompat tree; C) focus on subfamily Ornithogaloideae. The classification follows the World Checklist of Selected Plant Families. See E) for the legend of ancestral area reconstructions.

**Figure S3.** Biogeographic reconstruction on the PL-dated allcompat tree; D) focus on subfamily Urginoideae. The classification follows the World Checklist of Selected Plant Families. See E) for the legend of ancestral area reconstructions.

**Figure S3.** E) legend of ancestral area reconstructions.
