# Supplementary material for: Contrasting Biogeographic and Diversification Patterns in Two Mediterranean-Type Ecosystems
Source: PLoS One. 2012 Jun 20;7(6):e39377. doi: 10.1371/journal.pone.0039377 (PMC3379972; doi:10.1371/journal.pone.0039377)
Supplement: Table S1 — Classification and genera of Hyacinthaceae, with generic delimitation, distribution and species numbers. (DOC) [file pone.0039377.s005.doc]

**Electronic Supplementary Material**

**Contrasting biogeographic and diversification patterns in two Mediterranean-type ecosystems**

**Sven BUERKI^1,5,6^, Sarah JOSE^1,5^, Shrirang R. YADAV^2^, Peter GOLDBLATT^3^, John C. MANNING^4^, Félix FOREST^1,6^**

^1^Jodrell Laboratory, Royal Botanic Gardens, Kew, Richmond, Surrey, TW9 3DS, United Kingdom.

^2^Department of Botany, Shivaji University, Kolhapur-416 004(MS), India.

^3^B.A. Krukoff Curator of African Botany, Missouri Botanical Garden, PO Box 299, St. Louis, MO ^6^3166-0299, U.S.A.

^4^Compton Herbarium, Kirstenbosch Research Centre, South African National Biodiversity Institute, Claremont 7735, South Africa.

^5^ These authors contributed equally to this work and are considered co-first authors

^6^ Authors for correspondence: [s.buerki@kew.org](mailto:s.buerki@kew.org); [f.forest@kew.org](mailto:f.forest@kew.org)

**Table S1.** Classification and genera of Hyacinthaceae. Generic delimitation, distribution and species numbers are based on the World Checklist of Selected Plant Families with modifications . Distributions for the Cape of South Africa are from . Distributions: A, America; B, Madagascar; C, Sub-Saharan Africa; D, Cape of South Africa; E, Mediterranean Basin; F, Northern Europe; G, Middle East; H, Asia;. The first number in the distribution columns is the total number of species found in a given area and the second number is the number of species from this area that are included in the present study.

| **Subfamilies / Tribes / Genera** | **No. species (included)** | **Distribution** | **A** | **B** | **C** | **D** | **E** | **F** | **G** | **H** |
| --- | --- | --- | --- | --- | --- | --- | --- | --- | --- | --- |
| **Subfamily Oziroëoideae** |  |  |  |  |  |  |  |  |  |  |
| *Oziroë* Raf. | 5 (2) | SW Peru to southern South America | 5-2 |  |  |  |  |  |  |  |
|  |  |  |  |  |  |  |  |  |  |  |
| **Subfamily Ornithogaloideae** |  |  |  |  |  |  |  |  |  |  |
| *Albuca* L. | 154 (23) | Mainly sub-Saharan Africa, esp. winter-rainfall region, Madagascar, Arabian Peninsula |  | 1-1 | 144-24 | 36-12 |  |  |  |  |
| *Dipcadi* Medik. | 40 (15) | Africa, Madagascar, Socotra, Mediterranean to India |  | 2-1 | 24-5 | 4-2 | 3-2 |  | 4-2 | 11-7 |
| *Ornithogalum* L. | 181 (57) | Africa, Europe to Afghanistan |  | 1-1 | 76-27 | 28-17 | 74-26 | 32-11 | 41-8 | 2-0 |
| *Pseudogaltonia* (Kuntze) Engl. | 1 (1) | Namibia and Botswana |  |  | 1-1 |  |  |  |  |  |
|  |  |  |  |  |  |  |  |  |  |  |
| **Subfamily Urgineoideae** |  |  |  |  |  |  |  |  |  |  |
| *Bowiea* Harv. ex Hook.f. | 1 (1) | E Tropical Africa to South Africa |  |  | 1-1 | 1-1 |  |  |  |  |
| *Drimia* Jacq. ex Willd. | 120 (37) | Mediterranean to Myanmar, Africa |  | 9-3 | 80-21 | 28-11 | 13-8 |  | 1-1 | 6-4 |
|  |  |  |  |  |  |  |  |  |  |  |
| **Subfamily Hyacinthoideae** |  |  |  |  |  |  |  |  |  |  |
| Tribe Pseudoprospereae |  |  |  |  |  |  |  |  |  |  |
| *Pseudoprospero* Speta | 1 (1) | South Africa |  |  | 1-1 |  |  |  |  |  |
|  |  |  |  |  |  |  |  |  |  |  |
| Tribe Massonieae |  |  |  |  |  |  |  |  |  |  |
| *Daubenya* Lindl. | 8 (7) | Cape |  |  | 7-6 | 3-3 |  |  |  |  |
| *Drimiopsis* Lindl. & Paxton | 14 (5) | Tropical & South Africa |  |  | 14-5 |  |  |  |  |  |
| *Eucomis* L'Her. | 10 (5) | S Tropical Africa, South Africa |  |  | 10-5 | 3-1 |  |  |  |  |
| *Lachenalia* J.Jacq. ex Murray | 116 (8) | Namibia to Cape, Free State |  |  | 63-3 | 69-7 |  |  |  |  |
| *Ledebouria* Roth | 52 (8) | Tropical & South Africa, SW Arabian Peninsula, Madagascar, India, Sri Lanka |  | 1-1 | 51-7 | 4-1 |  |  | 1-1 | 1-1 |
| **Table S1 (cont.)** |  |  |  |  |  |  |  |  |  |  |
|  |  |  |  |  |  |  |  |  |  |  |
| **Subfamilies / Tribes / Genera** | **No. species (included)** | **Distribution** | **A** | **B** | **C** | **D** | **E** | **F** | **G** | **H** |
| **Subfamily Hyacinthoideae** (cont.) |  |  |  |  |  |  |  |  |  |  |
| Tribe Massonieae (cont.) |  |  |  |  |  |  |  |  |  |  |
| *Massonia* Thunb. ex L.f. | 13 (6) | South Africa |  |  | 13-6 | 5-5 |  |  |  |  |
| *Merwilla* Speta | 3 (1) | S Tropical Africa, South Africa |  |  | 3-1 |  |  |  |  |  |
| *Namophila* U.Müll.-Doblies & D.Müll.-Doblies | 1 (1) | Namibia |  |  | 1-1 |  |  |  |  |  |
| *Resnova* van der Merwe | 5 (3) | South Africa |  |  | 5-3 |  |  |  |  |  |
| *Schizocarphus* van der Merwe | 1 (1) | Tanzania to South Africa |  |  | 1-1 |  |  |  |  |  |
| *Spetaea* Wetschnig & Pfosser | 1 (1) | Cape |  |  |  | 1-1 |  |  |  |  |
| *Veltheimia* Gled. | 2 (1) | Cape |  |  | 2-1 | 2-1 |  |  |  |  |
|  |  |  |  |  |  |  |  |  |  |  |
| Tribe Hyacintheae |  |  |  |  |  |  |  |  |  |  |
| *Alrawia* (Wendelbo) K.M.Perss. & Wendelbo | 2 (1) | NE Iraq to Iran |  |  |  |  |  |  | 2-1 |  |
| *Barnardia* Lindl. | 2 (1) | Baleares, N Africa, China to Temperate East Asia |  |  |  |  | 2-1 |  |  | 1-1 |
| *Bellevalia* Lapeyr. | 65 (10) | Mediterranean to Central Asia |  |  |  |  | 37-6 | 2-0 | 35-5 | 4-2 |
| *Brimeura* Salisb. | 3 (1) | SW Europe |  |  |  |  | 3-1 |  |  |  |
| *Fessia* Speta | 11 (4) | Iran to Central Asia & Pakistan |  |  |  |  | 1-1 |  | 7-1 | 6-2 |
| *Hyacinthella* Schur | 17 (5) | E & SE Europe to North Iran |  |  |  |  | 13-5 | 3-1 | 5-0 |  |
| *Hyacinthoides* Heist. ex Fabr. | 11 (8) | W & SW Europe to NW Italy, NW Africa |  |  |  |  | 11-8 | 1-1 |  |  |
| *Hyacinthus* L. | 3 (2) | S Turkey to N Israel |  |  |  |  | 1-1 | 2-1 | 3-2 |  |
| *Leopoldia* Parl. | 10 (4) | Canary Island, Central Europe to Mediterranean & Iran |  |  |  |  | 10-4 | 2-2 | 6-4 |  |
| *Muscari* Mill. | 42 (5) | Europe, Mediterranean to Central Asia |  |  |  |  | 38-5 | 5-3 | 10-2 | 1-1 |
| *Prospero* Salib. | 13 (4) | NW Europe to Mediterranean, Hungary to Caucasus |  |  |  |  | 13-3 | 1-1 | 1-1 |  |
| *Pseudomuscari* Garbari & Greuter | 7 (6) | E Mediterranean to Iran |  |  |  |  | 2-1 | 3-3 | 5-4 |  |
| *Puschkinia* Adams | 2 (1) | SE Turkey to NW Iran |  |  |  |  | 1-0 |  | 2-1 |  |
| *Scilla* L. | 80 (18) | Europe, Macaronesia to Iran, Tropical Africa |  |  | 32-0 |  | 41-17 | 7-2 | 10-1 |  |
| *Zagrosia* Speta | 1 (1) | W Asia |  |  |  |  |  |  | 1-1 |  |
