# Supplementary material for: Contrasting Biogeographic and Diversification Patterns in Two Mediterranean-Type Ecosystems
Source: PLoS One. 2012 Jun 20;7(6):e39377. doi: 10.1371/journal.pone.0039377 (PMC3379972; doi:10.1371/journal.pone.0039377)
Supplement: Table S3 — Divergence time estimates and associated confidence intervals for selected nodes. (DOC) [file pone.0039377.s007.doc]

**Electronic Supplementary Material**

**Contrasting biogeographic and diversification patterns in two Mediterranean-type ecosystems**

**Sven BUERKI^1,5,6^, Sarah JOSE^1,5^, Shrirang R. YADAV^2^, Peter GOLDBLATT^3^, John C. MANNING^4^, Félix FOREST^1,6^**

^1^Jodrell Laboratory, Royal Botanic Gardens, Kew, Richmond, Surrey, TW9 3DS, United Kingdom.

^2^Department of Botany, Shivaji University, Kolhapur-416 004(MS), India.

^3^B.A. Krukoff Curator of African Botany, Missouri Botanical Garden, PO Box 299, St. Louis, MO ^6^3166-0299, U.S.A.

^4^Compton Herbarium, Kirstenbosch Research Centre, South African National Biodiversity Institute, Claremont 7735, South Africa.

^5^ These authors contributed equally to this work and are considered co-first authors

^6^ Authors for correspondence: [s.buerki@kew.org](mailto:s.buerki@kew.org); [f.forest@kew.org](mailto:f.forest@kew.org)

**Table S3.** Divergence time estimates and associated confidence intervals for selected nodes and comparison of nodal support from the Bayesian and maximum likelihood analyses.

|  |  | **Penalized likelihood inference** | | | **Node support** | |
| --- | --- | --- | --- | --- | --- | --- |
| **Classification** | **NodeID** | **Mean node age (My)** | **5% (My)** | **95% (My)** | **Bayesian posterior probability** | **Bootstrap support (%)** |
| MRCA Hyacinthaceae | 257 | 70.24 | 70.10 | 70.10 | 0.98 | 90 |
| MRCA Ornithogaloideae + Urgineoideae + Hyacinthoideae | 258 | 66.22 | 61.46 | 69.43 | 0.96 | 85 |
| MRCA Urgineoideae + Hyacinthoideae | 259 | 64.35 | 59.33 | 68.18 | 0.76 | 55 |
| Urgineoideae | 260 | 45.89 | 39.54 | 52.21 | 1.00 | 100 |
|  | 261 | 40.64 | 34.51 | 47.16 | 1.00 | 95 |
| Hyacinthoideae | 298 | 54.95 | 46.72 | 62.99 | 1.00 | 82 |
|  | 299 | 51.45 | 43.90 | 58.87 | 0.96 | 68 |
|  | 300 | 44.52 | 37.49 | 51.90 | 1.00 | 95 |
|  | 301 | 43.44 | 36.50 | 50.69 | 0.37 | 13 |
|  | 302 | 38.88 | 32.37 | 46.21 | 1.00 | 94 |
|  | 368 | 47.53 | 39.68 | 55.76 | 0.90 | 71 |
|  | 372 | 23.92 | 17.75 | 31.08 | 1.00 | 100 |
| Ornithogaloideae | 414 | 38.00 | 32.32 | 44.02 | 1.00 | 100 |
|  | 415 | 35.73 | 30.28 | 41.59 | 0.99 | 97 |
|  | 416 | 30.76 | 25.96 | 36.05 | 1.00 | 99 |
|  | 471 | 26.58 | 20.85 | 32.51 | 1.00 | 100 |
|  | 484 | 26.56 | 21.16 | 32.57 | 1.00 | 100 |
| Oziroeoideae | 511 | 31.23 | 20.22 | 43.83 | 1.00 | 100 |
